# Letter to editor on “Efficacy and safety of minor salivary gland transplantation for the treatment of severe dry eye disease: a single-arm meta-analysis”

**DOI:** 10.1097/JS9.0000000000003582

**Published:** 2025-10-03

**Authors:** Yinwen Shi

**Affiliations:** Department of Ophthalmology, Sichuan Taikang Hospital, Chengdu, China

*Dear Editor,*

We read with great interest the recent meta-analysis on minor salivary gland transplantation (MSGT) for severe dry eye disease (DED)^[1]^. The authors are to be commended for systematically synthesizing the limited evidence available for this rare and challenging condition. Their findings suggest that MSGT may offer meaningful symptomatic relief and objective benefits in patients with refractory DED. However, from a clinical perspective, several issues warrant further discussion before MSGT can be considered a broadly applicable treatment option. We declare that all of the following content complies with the TITAN guidelines and submit the guideline checklist^[2]^.

First, most included studies focused on patients with Stevens–Johnson syndrome (SJS), mucous membrane pemphigoid, or graft-versus-host disease, who often present with systemic immune dysregulation and extraocular mucosal involvement^[3,4]^. Donor gland function, oral mucosal health, and systemic disease activity may therefore strongly influence graft survival and secretory capacity. Unfortunately, most studies relied on gross inspection of donor sites without standardized preoperative functional assessment, raising concerns about patient selection criteria and the reproducibility of outcomes in real-world practice. In addition, symptomatic improvement was largely captured through patient interviews rather than validated dry eye questionnaires, limiting comparability across studies.

Second, safety outcomes deserve careful consideration. Complications such as lip hypoesthesia, cosmetic changes, and graft necrosis, while not life-threatening, may substantially affect quality of life – particularly in younger patients who already carry a significant psychosocial burden from systemic disease. Given the relatively short follow-up (mean 2.87 years) and small sample sizes, the reported low complication rates should be interpreted with caution. Whether MSGT can sustain efficacy and safety over decades remains uncertain for patients requiring lifelong management.

Finally, MSGT is a technically demanding microsurgical procedure performed only in specialized centers. In many regions, patients with severe DED already have limited access to corneal transplantation, scleral lenses, or biologic therapies – let alone MSGT. Thus, while the current meta-analysis provides encouraging data, future research should emphasize standardized donor assessment, validated outcome measures, long-term follow-up, and health economic evaluation to better define the role of MSGT within the therapeutic landscape of severe DED.

In summary, MSGT represents a promising surgical option for selected subgroups of refractory DED. Nevertheless, its clinical applicability outside tertiary centers remains constrained, and careful patient selection, standardized outcome reporting, and extended monitoring are essential before considering widespread adoption.

## Data Availability

Not applicable.
